# Texas Delegates to Pan-American Medical Congress

**Published:** 1893-08

**Authors:** 


PAN-AMERICAN MEDICAL CONGRESS—TEXAS DELEGATION.—The publication in the newspapers, of the Governor's list of delegaes to the Pan-American Medical Congress was premature. This was a list which had been submitted but had not been duly considered and selections had not then been made. Below we give the list of delegates to whom commission was issued by the Governor:

Dr. R. M. Swearingen, Austin,
Dr. W. M. Yandell, El Paso,
Dr. E. W. Lightfoot, Pittsburg,
Dr. A. M. Denman, Lufkin,
Dr. Joseph Kemp, Walnut,
Dr. H. M. Beaty, Plano,
Dr. J. P. Knox, Denton,
Dr. F. E. Pope, Honey Grove,
Dr. Wm. Boyd, Waxahachie,
Dr. Pat Clark, Ennis,
Dr. J. W. McLaughlin, Austin,
Dr. J. F. Y. Paine, Galveston,
Dr. T. D. Wooten, Austin,
Dr. A. M. Douglas, Covington,
Dr. F. Herff, San Antonio,
Dr. J. W. Gallagher, Graham,
Dr. R.H. Harrison, sr., Columbus,
Dr. W. J. Goodman, Tyler,
Dr. L. L. Whitaker, Elmendorf,
Dr. J. F. Eves, Millican,
Dr. J. L. Jones, Gatesville,
Dr. L. B. Roebuck, Italy,
Dr. H. A. Tutwiler, Flatonia.
Dr. J. M. Willis, Waco,
Dr. J. T. Wilson, Sherman,
Dr. Amos Graves, San Antonio,
Dr. E. L. Thompson, Dallas.
Dr. T. C. Osborn, Cleburne,
Dr. J. H. Sears, Waco,
Dr. Geo. Cupples, San Antonio,
Dr. T. J. Tyner, Austin.

Secretary West, of the Texas State Medical Association, informs us that no delegates were elected at last meeting, but Drs. Ed. Randall, A. J. Smith, David Cerna and H. A. West, of Galveston; Dr. M. M. Scott, of Brownwood, and Dr. J. D. Burch, of Aurora, will attend the Congress and have been duly commissioned as representatives of the State Association.

